# Using an Automated Evaluation System to Improve Medical Student Evaluation Response Rate and Reduce Administrative Burden

**DOI:** 10.1002/aet2.70257

**Published:** 2026-09-23

**Authors:** Chaiya Laoteppitaks, Alisha Agarwal, Jason Gonzalez, Nina Mingioni, Xiao Chi Zhang

**Affiliations:** ^1^ Department of Emergency Medicine Thomas Jefferson University Philadelphia Pennsylvania USA; ^2^ Sidney Kimmel Medical College Philadelphia Pennsylvania USA; ^3^ Department of Internal Medicine Thomas Jefferson University Philadelphia Pennsylvania USA

## Abstract

**Background:**

Email reminders facilitate timely task completion but can create substantial administrative burdens for clerkship staff. In our emergency medicine (EM) clerkship, third‐year medical students initiate digital evaluation requests after each clinical shift, requiring frequent manual reminders to maintain completion rates. The time‐intensive nature of this process highlights the need for a more efficient and scalable approach.

**Methods:**

This multi‐site, pre‐post study evaluated clinical performance evaluation completion rates and time to completion during the required EM clerkship over 48 weeks before and after implementation of an automated reminder system in September 2024. The system integrated existing survey functionality with Microsoft Excel and Microsoft Power Automate to track evaluation requests and completion and generate reminders every 2 days for up to 28 days. Primary outcomes were evaluation completion rate and time to completion. A theoretical cost analysis compared estimated personnel costs of the manual reminder process, based on administrator time and median salary estimates, with projected programming development and maintenance costs over 3 years.

**Results:**

A total of 5747 evaluations were sent during this period to attending and resident evaluators. Overall completion rate rose modestly post‐intervention (71.4% to 75.1%, *p* < 0.001), with attending evaluation rate demonstrating greater improvement from 73.9% to 81.6% (*p* < 0.001). Conversely, both resident (median 2.67 to 3.98 days) and attending completion time increased (median 2.59 to 2.74 days), *p* = 0.002. Based on the state salary estimates and our institutional experience, the automated approach could yield estimated net savings of $2850 in the first year and $9560 over 3 years.

**Conclusions:**

Automated reminders improved evaluation completion but did not improve timeliness of completion, which may not translate into meaningful formative benefits if feedback is delayed. Further studies should focus on the impact on cognitive burden and evaluation quality before large‐scale automated systems can be optimized and sustainably integrated into medical education.

## 1 Introduction

Medical students rely on verbal and written feedback from clinical preceptors to reflect, address deficiencies, and develop clinical competence. Written evaluations also contribute to clerkship grades and residency applications, with more than 80% of program directors rating them as “important” or “extremely important” [1]. Despite their importance, nearly half of students do not receive timely or substantive evaluations from their preceptors [2]. Evaluation completion may be limited by competing demands and the time required to provide feedback; for example, physicians spend approximately 7 min per written evaluation, with some requiring more than 30 min [3]. One study estimated that for every hour spent on direct clinical care, physicians dedicate two additional hours to paperwork and administrative responsibilities, including documentation, insurance claims, prescription refills, and prior authorizations [4]. With administrative tasks accounting for roughly 15%–30% of total U.S. healthcare expenditures [5], the ever‐expanding burden of indirect clinical duties has emerged as a major contributor of physician burnout, with one meta‐analysis citing that high workload was associated with a 2.42 times greater risk of burnout [6].

At our institution, the educational administrative team assumed responsibility in collecting student evaluations, but clerkship leadership is ultimately accountable for evaluation completion. As a result, the work required for sending repetitive follow‐up reminder emails reflects on the cumulative administrative burden shared by the entire clerkship team [7]. Given the growing use of automation in improving efficiency and reducing unnecessary resource utilization [8], we designed an automated email reminder system for evaluation requests initiated by Emergency Medicine (EM) clerkship students. We hypothesized this process would (1) increase evaluation completion rates and (2) provide a more cost‐efficient alternative for the institution over time.

## 2 Methods

This pre‐post multicenter study evaluated completion rates of clinical performance evaluations during the required Emergency Medicine (EM) clerkship one academic year (48 weeks) before and after implementation of an automated reminder system for incomplete evaluation requests. The EM clerkship is a mandatory 3‐week rotation in which third‐year medical students (MS3s) complete approximately 10 clinical shifts across one of seven academic clinical settings under the supervision of attending physicians and/or senior resident educators. Approximately 20 students rotate during each 3‐week block and are randomly distributed across sites with exposures to a variety of clinical environments and teaching faculty. Students were required to submit at least one standardized electronic evaluation request per shift to an attending or resident physician, regardless of clinical site.

Student evaluations were collected using Qualtrics, a FERPA‐compliant cloud‐based experience platform that enables survey creation, distribution, and analysis. Students were expected to initiate the evaluation process independently by first submitting the evaluation request, which entails the details of their shift encounter to their evaluator. This action will generate a learner‐specific link to the assigned evaluator and, after the evaluation is completed, Qualtrics will immediately send an automatic, de‐identified receipt with all documented summative and constructive feedback back to the student. All requests and completed evaluations are recorded digitally in a FERPA‐compliant Microsoft Excel spreadsheet.

At our institution, the clerkship administrator was tasked to identify incomplete evaluations at the end of each week and send reminder emails. Subsequent escalation reminder emails were sent by the clerkship director, often involving additional stakeholders such as program directors, the Dean's office, Vice Chairs of education, and even departmental chairs, to encourage completion. In our experience, the initial email‐based process required approximately 12 h per month of administrator effort, excluding phone calls, text messages, and video conferencing for unresolved cases, which were delegated to the clerkship director.

In the summer of 2024, the process of sending reminders for incomplete evaluations became automated. We hired our former clerkship director [CL], who has academic experience in EM clerkship requirements and evaluation metrics, as well as programming expertise in web development, Java, and JavaScript, to develop an automated system that tracks and sends reminders to delinquent evaluators. The automated process integrates Qualtrics automated email functionality with Microsoft Excel and Microsoft Power Automate (PA) Cloud Flows. When a student sends an evaluation request, a PA Cloud Flow automatically logs the request in Excel. Upon submission, the spreadsheet is updated in real time. The system identifies incomplete evaluations less than 28 days old and generates automated email reminders to the evaluator every 2 days until the evaluation is completed or 28 days have elapsed since the initial request. One month prior to implementation, the automation process was communicated to EM site directors for dissemination to their respective preceptors.

The primary outcomes were evaluation completion rate and time to evaluation completion among attending and resident physicians during the 48‐week periods before and after implementation of the automated reminder system. Evaluation completion rates were analyzed using logistic regression, while time to completion was analyzed using median regression. All statistical analyses were performed using Jamovi (Version 2.7.31). The secondary outcome was a cost analysis comparing the estimated administrative costs of weekly manual reminders with the projected costs of programmer development and maintenance of the automated system over 3 years. The theoretical cost of supporting student evaluation completion was estimated based on the time required by the clerkship coordinator (calculated as hours spent per month divided by a 40‐h work week) and the computer programmer (time spent developing, maintaining, and troubleshooting the program). Median salaries for each role in 2025 were estimated to be $63,569 ($30.6/h) and $82,125 ($39.5/h), respectively [9].

## 3 Results

The automated feedback reminder system was implemented in September 2024. During the 48‐week period preceding implementation (Pre‐Automation), 2747 evaluations were sent, including 1209 to attendings and 1538 to residents. During the 48‐week post‐intervention period (Post‐Automation), 3000 evaluations were sent, including 1380 to attendings and 1620 to residents. Among the total 5747 evaluation requests analyzed, the overall completion rate rose modestly post‐intervention (71.4% to 75.1%). However, there was a statistical difference among training levels: the attending completion rate increased substantially (73.9% to 81.6%), while the resident completion rate remained essentially unchanged (69.4% to 69.6%), a difference confirmed by logistic regression (Period × Training Level interaction, *p* < 0.001). Conversely, among completed evaluations, resident completion time increased (median 2.67 to 3.98 days), while the attending completion time changed minimally (median 2.59 to 2.74 days); median regression confirmed this difference was statistically significant (*p* = 0.002) and not attributable to outliers. Detailed breakdowns of completion rates are presented in Table 1.

**TABLE 1:** Student evaluation completion rate and time, 48‐weeks before and after automation process (Pre‐ and Post‐Automation).

Period	Training level	Evaluation sent	Evaluation completed	Completion rate (%)	Median completion time (days)
Pre‐Automation	Attending/fellow	1209	893	73.9%	2.59
	Resident	1538	1068	69.4%	2.67
	Combined	2747	1961	71.4%	2.64
Post‐Automation	Attending/fellow	1380	1126	81.6%	2.74
	Resident	1620	1128	69.6%	3.98
	Combined	3000	2254	75.1%	3.30
Total		5747	4215	73.3%	

*Note:* Logistic model for predictors of evaluation completion: (Pre‐ vs. Post‐Automation) × training level (attending vs. resident): *p* < 0.001; median regression for predictors of completion time among completed evaluations: *p* = 0.002.

Based on the state salary estimates and our institutional experience, our clerkship coordinator typically dedicated 12 h per month sending individual reminder emails, which would account for $367 per month, $4404 per year, and $13,212 over 3 years. In comparison, the initial cost to develop the automated system was estimated to be $474 (12 h × $39.5/h) and required approximately 2 h per month for maintenance ($79). While this would result in a larger year‐1 cost for starting the automation process ($474 + $79 × 12 = $1421), the cost to maintain this system would only be $948 annually and only cost $3317 over 3 years. This approach yields projected net savings of $2850 in the first year and $9560 over 3 years.

## 4 Discussion

### 4.1 Improved Student Evaluation Rate

Student evaluations are essential for providing formative feedback and assessing clerkship performance. Prior to automation, the administrative team relied on personalized reminders via email, text message, and in‐person requests, resulting in completion rates of 73.9% among attendings and 69.4% among residents. Following implementation of the automated reminder system, completion rates increased by 7.7% among attendings and 0.2% among residents.

The greater improvement among attendings may be related to differences in evaluation burden as well as the culture surrounding faculty incentives or repercussions of not completing evaluations. Residents consistently received more evaluation requests than attendings during both the Pre‐ and Post‐Automation periods (27% and 17% greater, respectively). Because the automated system was primarily designed to address the behavioral barrier of overlooking an incomplete evaluation, its effectiveness may be limited when noncompletion is driven by competing demands or a greater volume of outstanding requests. Residents may be particularly susceptible to this effect as they balance evaluation requests with clinical responsibilities, documentation, and educational activities.

Attendings, in contrast, may have fewer competing documentation demands related to learner evaluations and may be more responsive to automated prompts within their established educational responsibilities. Since completing evaluations may be considered as a performance metric for academic faculty at many institutions, these reminders may have helped faculty meet these existing expectations by prompting completion of outstanding evaluations and improving their overall metrics [10].

### 4.2 Increase Time to Evaluation Completion

While the study yielded overall improved completion rates, median time to evaluation completion increased following implementation of the automated reminder system, from 2.59 to 2.74 days among attendings and from 2.67 to 3.98 days among residents. Since all evaluators were notified of this new reminder system, the intervention may have reduced the perceived urgency to complete an evaluation immediately. This effect may have been more pronounced among residents, who may face greater competing clinical and educational demands and therefore be more likely to postpone evaluation completion until a later opportunity.

An alternative explanation is that automation enabled completion of evaluations that previously would have remained incomplete. Consequently, the Post‐Automation period may have included a greater proportion of evaluations completed after a longer delay, increasing the observed median time to completion while simultaneously improving the overall completion rate. Because the study did not directly assess evaluators' perceptions or reasons for delayed completion, these explanations remain speculative. Nevertheless, the findings suggest a potential unintended consequence of automated reminders: while repeated prompts may reduce the likelihood that evaluations are permanently overlooked, they may also create an opportunity for task deferral and delay the timely delivery of formative feedback to learners.

### 4.3 Reduction in Costs

Beyond the direct benefits to students, the implementation of automation also demonstrated potential improvements in time management and administrative efficiency. Prior to automation, the clerkship coordinator devoted approximately 12 h per month to managing reminder emails, representing a cost burden of $13,212 over 3 years. Although the initial startup expense of the automated system exceeded the monthly costs incurred with manual reminders, ongoing maintenance expenses were substantially lower, yielding nearly $10,000 in savings over the same period. These estimates likely underestimate the total administrative burden of manual follow‐up, as they do not include the time required by the clerkship director to personally identify and solicit outstanding evaluations from faculty and residents.

The scalability of the automated system also represents an important operational advantage. During a 12‐week period, the system generated more than 2000 automated reminders, a volume that would have required substantial administrative effort if completed manually. Assuming a minimum of 5 min per manual reminder—including identification of outstanding evaluations, message preparation, and follow‐up—this volume would represent approximately 167 h of administrative effort. Although this represents a projected rather than directly measured time savings, the findings demonstrate the capacity of automation to substantially reduce repetitive administrative tasks while maintaining a consistent reminder process. By shifting routine follow‐up from personnel to an automated workflow, administrative resources may be redirected toward higher‐value activities, including learner support, curriculum development, and faculty engagement.

### 4.4 Implications

This pilot study reflected the feasibility of incorporating an automated process to reallocate administrative resources, which could be particularly valuable in clerkships with large numbers of learners, evaluators, and clinical sites, where manual follow‐up is difficult to sustain. This study also identified a notable behavioral response to automation between the two most common student evaluators within the clinical environment: attending vs. resident physicians. Automation may be less effective when noncompletion reflects competing clinical responsibilities or a high volume of evaluation requests and may even result in greater burnout and email fatigue. Finally, the increase in time to completion represents a meaningful portion of the rotation during which students may not receive feedback on their performance. Delayed feedback may limit students' ability to recognize and address areas for improvement while they still have sufficient time to apply that feedback during the rotation. Thus, while automated reminders will help with the summative evaluation process by increasing the completion rate, it may not translate into meaningful formative improvements if the feedback was not delivered with sufficient timeliness.

## 5 Limitations

This study has several limitations. First, the automated system was developed and implemented by the former clerkship director, who possessed both the technical expertise and institutional knowledge required to design and iteratively refine the workflow. This dual role may limit the generalizability of the implementation process, as institutions without comparable technical expertise or familiarity with their evaluation infrastructure may require greater development time or external resources. Conversely, the ability to leverage existing institutional systems and prior experience with Qualtrics and cross‐platform integration likely reduced the cost and time required for implementation in our setting.

Second, the estimated cost savings were based on the administrative effort and commercially available labor costs at our institution in Pennsylvania rather than previously published cost models. These estimates may not fully capture the resources required for implementation, including unanticipated troubleshooting, system modifications, or deviations from existing evaluation software. Such technical requirements could necessitate additional programming and information technology support, potentially increasing both implementation and maintenance costs beyond those estimated in this study.

Third, the study evaluated completion rates and time to completion but did not assess the quality, specificity, or educational utility of the feedback provided. Increased completion does not necessarily equate to improved feedback. Automated reminders could increase the number of submitted evaluations while having little effect on the quality or usefulness of narrative comments. Future studies should therefore examine whether automation influences the content and quality of feedback, particularly whether increased evaluation volume is accompanied by meaningful narrative feedback.

Finally, evaluator perceptions of the automated reminder process were not assessed, limiting our ability to determine its acceptability or generalizability to other institutions. In our institution, students independently initiated digital evaluation requests after each clinical shift, rather than a composite evaluation. This workflow, along with the volume of automated reminders, may have contributed to notification fatigue or perceived administrative burden. Future implementation studies should explore evaluator‐reported measures of workload, acceptability, and satisfaction to assess the broader applicability of automated reminder systems.

## 6 Conclusions

Automated reminders may reduce reliance on manual follow‐up and improve completion but may be insufficient to overcome workload‐ or workflow‐related barriers. Complementary strategies, particularly for residents, such as integrating evaluation completion into existing clinical workflows, may be considered to improve response time and rates. Future research should qualitatively examine how evaluators perceive and respond to evaluation requests, including the impact on cognitive burden and evaluation quality before large‐scale automated systems can be optimized and sustainably integrated into medical education.

## Author Contributions

**Chaiya Laoteppitaks:** formal analysis, investigation, writing – original draft, writing – review and editing, methodology. **Alisha Agarwal:** writing – original draft. **Jason Gonzalez:** writing – review and editing, project administration. **Nina Mingioni:** supervision, methodology, investigation, writing – review and editing. **Xiao Chi Zhang:** supervision, formal analysis, project administration, methodology, writing – review and editing, writing – original draft, investigation, conceptualization.

## Funding

The authors have nothing to report.

## Conflicts of Interest

The authors declare no conflicts of interest.

## Data Availability

The data that support the findings of this study are available from the corresponding author upon reasonable request.
